# Using the resilience theory to understand and address migrant pandemic precarity among South African migrant populations

**DOI:** 10.1186/s13690-025-01573-9

**Published:** 2025-04-03

**Authors:** Ferdinand C. Mukumbang, Babatope O. Adebiyi

**Affiliations:** 1 Department of Global Health, School of Public Health, University of Washington, Seattle, USA; 2 Centre for Interdisciplinary Studies of Children, Families and Society, University of the Western Cape, Cape Town, South Africa; 3 Section of Rheumatology, Department of Paediatrics, Cumming School of Medicine, University of Calgary, Alberta, Canada

**Keywords:** Migrant pandemic precarity, COVID-19, Undocumented migrants, Adversity, Phenomenological interpretive analysis, South Africa

## Abstract

**Introduction:**

“Migrant pandemic precarity” describes the specific consequences and vulnerabilities experienced by migrants during the COVID-19 pandemic. Despite their precarity, migrants adopted some resilient behaviors. Utilizing the resilience theory, our study explored how migrants in South Africa managed to cope with heightened vulnerabilities during the COVID-19 pandemic and how these resilient behaviors can provide insights into addressing the health inequities experience by this population.

**Methods:**

We conducted an interpretive phenomenological analysis study to understand the key challenges of migrant populations in South Africa during the COVID-19 pandemic (2019–2022) and how resourceful they became in overcoming these challenges. Using a purposive sampling approach, we conducted 20 semi-structured interviews with migrants from other African countries, including asylum seekers, refugees, permit holders, and undocumented migrants in two South African provinces.

**Results:**

Three interconnected aspects of migrant pandemic precarity were revealed: financial insecurities, food insecurities, and health concerns. Social connectedness and resource provision ensured inclusivity and supported these migrant populations in navigating the difficulties posed by migrant pandemic precarity.

**Conclusions:**

The South African government should implement migrant-inclusive approaches and empower structures and programs that enhance migrants’ resilience to future crises. We argued that to reduce health inequities among migrant populations in South Africa, these resilience approaches can be harnessed in three ways. (1) the South African government should create mechanisms and processes to identify and integrate migrants with critical skills into their workforce. (2) enhancing collaborations between civil society organizations, local governments, and international organizations, such as the International Organization for Migration, to address food insecurities among the migrant population. (3) enforcing their constitutional mandate to provide free basic health care services to all migrants in South Africa by removing barriers such as health care provider attitudes toward migrants’ access to health care services.

**Supplementary Information:**

The online version contains supplementary material available at 10.1186/s13690-025-01573-9.

**Table:** 

**Text box 1. Contributions to the literature**
• Migrant pandemic precarity, the specific consequences and vulnerabilities experienced by migrants during the COVID-19 pandemic, received comparatively lesser attention.
• Using the resilience theory, we showed how migrants in South Africa address migrant pandemic precarity.
• Collaborations between civil society organizations, local governments, and international organizations can enhance migrant resilience.

## Introduction

“It’s your reaction to adversity, not adversity itself, that determines how your life’s story will develop.” **(Dieter F. Uchtdorf**).

International migrants face various challenges, including violence, exploitation, abuse, socio-economic hardships, and health issues, making them one of the most vulnerable groups globally [1]. Numerous studies have demonstrated that these vulnerabilities were further exacerbated during the COVID-19 pandemic [2–4]. Scholars have referred to the unique consequences and vulnerabilities experienced by migrants during the COVID-19 pandemic as “migrant pandemic precarity” [5, 6].

South Africa has the highest number of migrants on the African continent [7]. It has been argued that South Africa’s COVID-19 pandemic response allowed the government to further its unfriendly agenda towards migrants [8, 9]. The pandemic provided an excuse for the government to push through xenophobic policies that were already in the works [10]. Under this xenophobic-rhetoric clout, we believe that the precariousness of migrants during the pandemic is heightened, and it becomes challenging to tap into their resilience.

The concept of migrant pandemic precarity undergirds the distinct impacts of the COVID-19 pandemic on migrants [11]. Besides the immediate triggers of the pandemic, this precarity has been influenced by past and present state policies and the historically evolved socio-economic structure [6]. Therefore, the precarious situation faced by migrants during the COVID-19 era is rooted in long-standing systemic inequality and inequity drivers that have been enforced, re-emerged, and adapted [12]. Due to these pre-existing inequities, migrants experienced limited access to COVID-19 prevention measures, disproportionate unemployment, inadequate living conditions that exposed them to outbreaks, stigma and discrimination, uncertainty regarding their legal status, and housing challenges, conditions that worsened and exacerbated their vulnerabilities [13, 14].

The term “migrant precarity” encompasses various human conditions related to employment, legal status, housing situations, and access to societal resources [15]. The United Nations [16] categorizes the precarity and vulnerabilities of migrants into three main areas: health, socio-economic, and protection crises. Health crises encompass unsanitary living conditions, limited access to healthcare services, and food insecurities. Socio-economic crises involve increasing unemployment rates, loss of livelihoods, and decreased remittances. Finally, the protection crisis pertains to challenges in accessing asylum and detention, as well as forced returns and deportations.

Migrants often face weakened social support networks, limited socio-economic opportunities, unequal access to healthcare and social services, unstable housing conditions, uncertain living and working environments, and an increased risk of exploitation and abuse [17]. During the COVID-19 pandemic, the vulnerabilities of these migrants were exacerbated as they faced a higher risk of severe outcomes and mortality compared to the general population [17, 18]. Despite this precarity, many migrants worldwide showed resilience [13, 19]. In this context, resilience refers to coping with stressful events characterized by social competence, problem-solving skills, autonomy, and positive expectations for the future [21]. Historically, migrants have come together to seek recognition, rights, dignity, and justice, overcoming their vulnerabilities [20].

Different conceptualizations of resilience can be found in the literature [21]. This paper examines resilience as coping strategies or experiences migrants adopt to overcome the precarity they faced during COVID-19. Specifically, we focus on how migrants sustain their well-being in the face of these challenges. While some authors have separately explored migrant precarity and resilience during the pandemic, very few have investigated how migrants maintained their well-being despite the precarity [14]. A cursory search of the literature using Google Scholar yielded no papers investigating the resilience of migrants during the COVID-19 pandemic using resilience theory. Therefore, we aim to explore how migrants in South Africa mobilized to mitigate the precarities they experienced during the implementation of lockdown measures, drawing on resilience theory.

## Methods

### Study design

We conducted a study using interpretive phenomenological analysis (IPA), a qualitative research approach that examines how individuals interpret and make sense of their major life experiences [22]. Ontologically, IPA recognizes multiple meaningful representations of human experiences [23]. The primary focus of IPA is to understand how participants interpret and make sense of their experiences [24]. We found that qualitative methods, such as IPA, were suitable for understanding individuals’ experiences during the COVID-19 pandemic. They provide insights into the evolving situation and how individuals effectively manage these unprecedented circumstances [21].

### Study setting

The study was conducted in the Gauteng and Western Cape provinces, the largest and third largest South African provinces respectively [25]. South Africa has one of the highest rates of cross-border migration, with an estimated 1.02 million people immigrating between 2016 and 2021 [26]. During this period, the Gauteng and Western Cape provinces received the highest number of immigrants due to their better cultural, economic, environmental, and socio-political conditions than the other seven provinces [26].

The COVID-19 pandemic containment measures implemented by the South African government, such as the national lockdown, exacerbated the unequal treatment of migrant populations in the country [27]. For example, to address the hunger crisis faced by disadvantaged South Africans, the government received various charitable donations and provided official support in the form of food parcels. While civil society organizations working with migrants have made efforts to support refugees and asylum-seekers during the lockdown, there has been little support or consideration from the South African government [27]. Moreover, migrant populations in South Africa have limited access to healthcare services, including testing and treatment for COVID-19. Therefore, our objective was to investigate the migrant pandemic precarity and their medicating process based on the experiences of migrant populations in South Africa.

### Conceptual framework

**Fig. 1:**
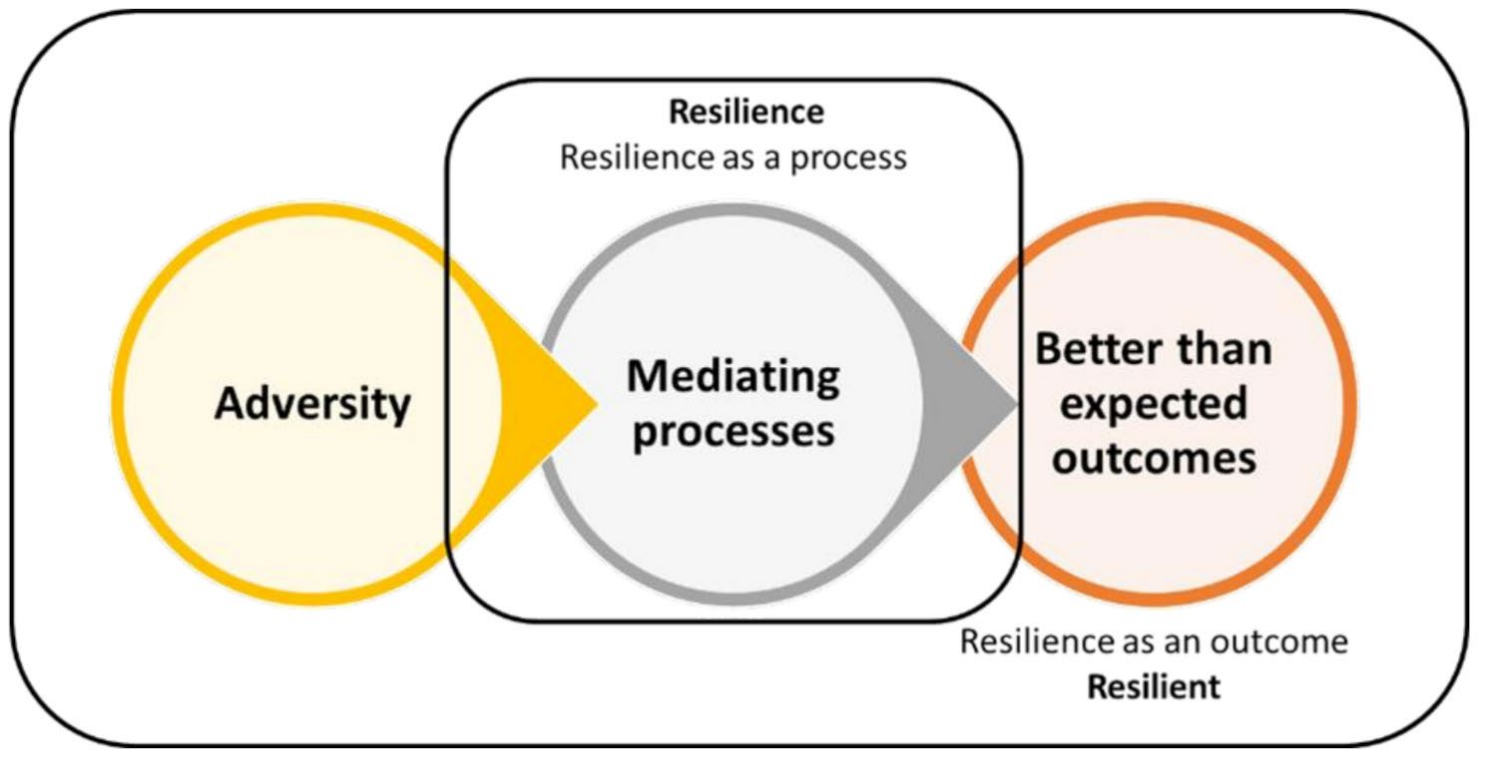
The resilience theory framework

The resilience theory underpinned this study as a conceptual framework. Resilience refers to strategies of endurance that people adopt to facilitate their day-to-day living but that do not change the circumstances that make their lives difficult [28]. The resilient theory, therefore, helps us understand how certain individuals can recover from difficult situations by focusing on their strengths [29]. Resilience is not just a personality trait but a dynamic process or system that effectively allows individuals to adapt to life’s challenges [23]. It encompasses three main processes: adversities, mediating processes, and outcomes surpassing expectations - Fig. 1 [30].

Van Breda [30] explained that patterns of adversity can be chronic or acute, explaining that chronic adversity extends over a considerable period and may have a pervasive impact on a person’s life. While the COVID-19 pandemic was a transient event, we considered most of the adversities experienced by the migrants to be acute. Nevertheless, experiences such as discrimination and health inequities are chronic as they are informed by other systemic issues, notably xenophobia [31]. At the heart of the resilience construct are the mediating processes (also referred to as resilience processes or protective resources), which enable people to achieve better-than-expected outcomes when they experience adversity [32, 33].

Resilience researchers have brought about a paradigm shift in developmental research by replacing a deficit-based approach, which focuses on identifying risk factors and vulnerabilities, with a strength-based approach. This new approach examines the positive variables contributing to favorable outcomes [30, 31, 34]. These researchers argue that there are three protective factors for adaptive systems: individual, family, and community. However, they believe most protective factors originate outside the individual [35]. The resilience theory explains how to adapt well to adversity, trauma, tragedy, threats, and stress [36]. In light of the COVID-19 pandemic, migrants faced various adversities (precarity) and employed certain mediating processes to foster resilience.

### Participant selection

Purposive and snowball sampling were used to recruit study participants, including asylum seekers, refugees, permit holders, and undocumented migrants. We aimed to recruit diverse participants based on family structure, gender, age, socio-economic status, culture, and employment status. Ultimately, a final sample of 20 participants provided in-depth insights into the current challenges experienced by families during the pandemic.

### Inclusion/Exclusion criteria

Participants in the study needed to meet the following criteria: (1) Between 18 and 64 years old, (2) have migrant, asylum-seeking, or refugee status. (3) They had to be from a sub-Saharan country, except South Africa and (4) be able to communicate in either French or English. Participants aged 18 to 65 were included to avoid consent issues and potential exploitation, as migrants are a vulnerable population. Minors were excluded due to their increased vulnerability. Our focus is on migrants from sub-Saharan countries, as they are the most alienated population in South Africa, facing xenophobic attacks and unfair treatment. Migrants from other African countries and continents were excluded due to comparatively less xenophobic alienation. Asylum-seeking or refugee status was critical for inclusion, as the study focuses on their COVID-19 vaccination experiences. Francophonie African migrants were also included to capture varied perspectives as they face language barriers and interaction issues in South Africa.

Participants were recruited using two methods: direct contact and snowballing. For direct contact in the Western Cape Province, we contacted migrant populations living in Cape Town using our research team’s contact list. Additionally, we posted an advertisement on the Facebook page of the African Centre for Migration & Society to attract participants. The initial participants obtained through these methods were then asked to invite other migrant populations in Cape Town with whom they were acquainted. When contacting potential participants, we followed a specific process through which eight participants were recruited. It is worth noting that the Facebook recruitment approach has successfully recruited participants for studies on social injustices [37]. Before each interview session, the participants were reminded of the study aim and their role and consequently requested to sign an informed consent before participation. Table 1 below provides an overview of the participants’ characteristics.

Recruitment in Gauteng was more direct and was done through The Tshwane Migrant Project. The Tshwane Migrant Project started on July 12, 2019, to provide a supportive, specialized HUB for undocumented migrants, asylum seekers, permit holders, and refugees living in Tshwane. The HUB offers free and confidential primary health care services, mental health care, and referrals for secondary or specialized care to people who are often unable to access appropriate health care or social services. Participants were recruited via two avenues: direct contact and snowballing. For direct contact, we recruited a research assistant who worked as the Health Promotion Supervisor at Médecins Sans Frontier, Tshwane Migrant Project, who recruited participants through the project. For the snowballing component, participants recruited by the research assistant were then asked to suggest other migrants who met the study criteria and would like to take part. After submitting the research proposal and obtaining ethics clearance from the University of the Western Cape ethics board, we received permission from the Tshwane Migrant Project to conduct the research in their organization. The research assistant presented the study aims and objectives to the migrants living at the HUB at the Sediba Hope Medical Centre in Tshwane CBD and requested that those interested in the study consent to participate by signing an informed consent. Twelve participants volunteered to take part in the study through the project.

**Table 1:** Participant characteristics

Characteristics	Participants (*N* = 20)
**Gender**	
Male	12
Female	8
**Country of Origin**	
Tanzania	1
Malawi	3
Rwanda	2
Congo DRC	5
Zimbabwe	6
Cameroun	1
Somalia	1
Nigeria	1
**Immigration status**	
Asylum seeker	11
Undocumented	5
Permit holder	3
Refugee	1
**Qualification**	
Masters	3
Degree	5
High school	9
Primary school	3
**Age**	
20–30	6
31–40	5
41–50	9
**Occupation**	
Student	5
Shop assistant	1
Unemployed	7
Technician	1
Trader	5
Community health worker	1

### Procedure: data collection

Data collection in IPA is to enable participants to provide a comprehensive account of their experiences and perceptions. Smith and Osborn [24] recommend using semi-structured interviews for data collection in an IPA study. Our data collection was done during phase three of COVID-19 restrictions. These restrictions allowed people to meet in public places but must wear a cloth face mask or a homemade item that covers the nose and mouth or another appropriate item to cover the nose and mouth [38]. We conducted face-to-face, in-depth interviews with 18 of the 20 research participants and WhatsApp interviews with two participants. In compliance with the level three COVID-19 restrictions, for the face-to-face interviews, we complied with all the social distancing regulations that were put in place at the time. Participants were free to choose the location of the interviews, including university study and social spaces, workplace offices, business centers, and the HUB.

We piloted the interview guide among five migrants (two from the Western Cape and three from Gauteng province). The pilot participants were not included in the final study sample. We used a pretested interview guide to ensure consistency among respondents and to align with the research aims and the central constructs of the resilient theory. The questions covered the following topics: (1) their experiences during the COVID-19 outbreak and the lockdown measures that were put in place following the outbreak, (2) the nature of the challenges that they faced, (3) what they did to try to overcome the challenges identified, (4) what were the outcomes of their efforts to overcome those challenges, and (5) what they would do if in similar situations again.

A research assistant conducted the interviews in the Gauteng Province, while the authors conducted the interviews in the Western Cape Province between July and September 2021. The interviews were in either French or English, depending on the participant’s preference. Each interview lasted between 30 and 45 min per participant or family unit. Permission was obtained from each participant to record the interview sessions, and a transcription specialist transcribed each session verbatim. The research assistants reviewed all the transcriptions for accuracy and made the necessary corrections or adjustments. The two interviews in French were transcribed verbatim and translated into English by the transcription specialist, who was fluent in both languages. All transcripts were de-identified using pseudonyms and prepared for analysis.

### Rigor and trustworthiness

A piloted interview guide was used in this study. The guide was tested with five participants and revised based on their feedback. After piloting the interview guides, we realized that some of the questions were ambiguous in meaning and had to be reformulated for clarity. The questions on what they did to address or remedy their situation were broad and required sub-questions on the different aspects of precarity for the information to be clearly articulated by the participants. The interviews were conducted by authors and research assistants with extensive experience in qualitative interviews with vulnerable populations.

To ensure rigor, we detailed the sampling, data collection, and analysis processes. Two investigators participated in the data analysis process, engaging in discussions to resolve coding differences and identify emerging themes. Coding was done independently using a selection of transcripts, and a codebook was developed through an iterative approach. The codebook was further revised based on additional transcript coding.

Researchers must “bracket” their preconceptions in phenomenological studies to ensure trustworthiness. Bracketing helps prevent preconceptions from influencing the data collection and interpretation processes. By avoiding subjectivity, we aim to capture the authentic meaning of the research respondents’ accounts [39]. Throughout the research, BOA and FCM managed their preunderstandings by discussing and writing down their experiences during the COVID-19 pandemic and how they navigated their challenges. Reflexivity was a part of the codebook development process.

### Data analysis

We conducted a deductive-inductive hybrid thematic data analysis. The deductive-inductive hybrid approach allows the researchers to lean on pre-existing theories and frameworks to guide their studies while generating or developing new insights from the data through the inductive approach [40]. Deductively, our analysis was guided by the three constructs of the resilience theory: adversity (precarity), mediating process, and better-than-expected outcome. Our analysis was inductive. Within these three constructs, we allowed the different themes within the construct to emanate from the data.

Data analysis in IPA requires the investigator to establish an interpretative relationship with the transcripts. To achieve this, we used thematic analysis grounded in the subjective lived experiences of the individuals [41]. The IPA process consisted of six stages: (1) Familiarizing ourselves with the data through repeated reading of the transcriptions. (2) Identifying the researcher’s orientation and potential bias - phenomenological reduction. (3) Identifying significant experiences and relationships of the migrants regarding COVID-19 vaccine uptake. (4) Identifying emerging themes by considering both micro-level data and macro-level interpretation. (5) Grouping themes and identifying overarching emerging themes [41].

FCM and BOA conducted data analysis. They individually read the transcripts to familiarize themselves with the unique narratives of the participants. Subsequently, they met to discuss the narratives, identifying significant experiences and perceptions of the migrants regarding COVID-19 vaccine uptake. They also openly discussed and documented their respective biases. Both authors developed a codebook based on the overarching resilient theory and the preliminary inductive analysis of the transcripts. BOA used the codebook to code the remaining transcripts. Discursive meetings were held with FCM to identify emerging themes that considered both micro-level data and macro-level interpretations. They collaborated on clustering the emerging themes to identify overarching themes.

## Results

The data revealed three main adversities: socio-economic, food insecurity, and health crises. Using resilience theory, we unpacked these aspects of the migrant pandemic’s precarity and how the migrants went about mitigating the effects of these adversities to obtain better than expected outcomes in each aspect.

### Socio-economic crisis as migrant precarity and resilience

**Fig. 2:**
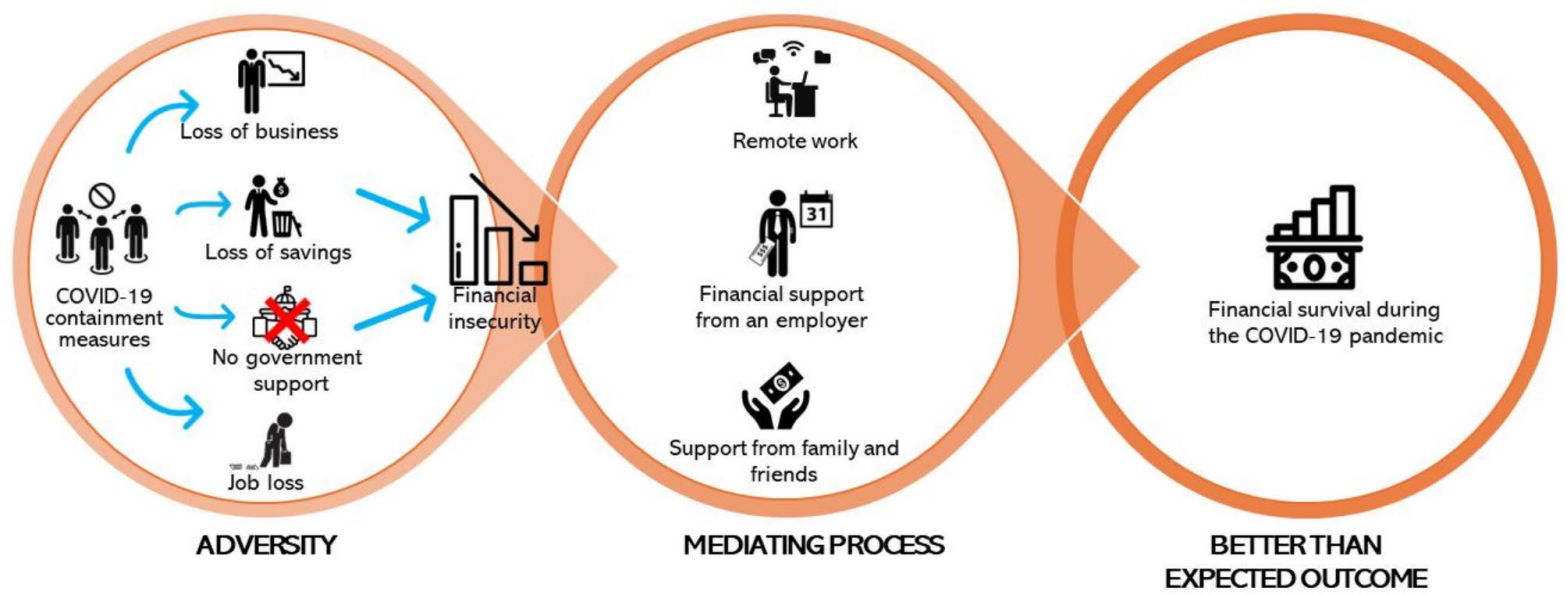
A resilient theory conceptualization of socio-economic crisis based on emerging data

The socio-economic crisis expressed itself as financial insecurity, one of the central adversary migrants faced during the COVID-19 pandemic. Financial insecurity occurred through four mechanisms: (1) Loss of business, (2) loss of employment, (3) depletion of savings, and (4) Lack of support. Figure 2 illustrates how migrants survived the financial hardship with the resources obtained from various sources.

Figure 2 shows how our data connects the three constructs of the resilient theory. The respondents illustrate how, through the COVID-19 containment measures, migrants in South Africa found themselves in a socio-economic crisis. The respondents identified that financial precarity was ushered through loss of job, savings depletion, business loss due to lack of sales, and being sidelined from South African government support. Migrants tried to overcome their socio-economic precarity by engaging in or retaining remote work and receiving financial support from their employers, families, and friends. Owing to these measures, most of these migrants could survive the COVID-19 pandemic era.

#### Socio-economic crisis

Migrants reported losing their livelihood through the loss of jobs during the COVID-19 pandemic.

I was working before, and they closed when the lockdown started, and I did not get the job again **[Voice 7; Zimbabwe; Undocumented**,** GP].**

Before the lockdown, I was working when we announced on the televisions [SIC] that we should no longer leave the houses because of COVID [SIC] (**Voice 13; Congo DRC; Asylum Seeker**,** WCP)**.

Those involved in petty or illegal trading also reported losing business opportunities because of the COVID-19 pandemic and the preventative rules.

Now I am not working. I am not selling the vegetables. Metro police took all the stuff where I was selling vegetables. Since then, I have not known when I will return to sell. They said no, no, do not sell here. And now I think I am not working **(Voice 5; Malawi; Asylum Seeker**,** GP)**.

The study participants indicated that savings depletion occurred during the COVID-19 pandemic lockdown. Many individuals, including migrants, ultimately used their savings and business capital.

I think my savings are finished. It has happened because it is only one week, they say (lockdown), since the lockdown started. Because it is only a small amount of money, I must know maybe to buy food for the day **(Voice 5; Malawi; Asylum Seeker**,** GP)**.

I was saving money, but I have two sisters and am paying their school fees. The money [saving] is finished **(Voice 7; Zimbabwe; Undocumented**,** GP)**.

Many participants did not receive the required assistance, making the COVID-19 pandemic lockdown difficult.

Like me? I did not get any assistance from anyone I was assisting myself **(Voice 4; Zimbabwe; Undocumented**,** GP)**.

Some were getting [assistance], but personally, in my household, I did not get **(Voice 1; Tanzania; Permit Holder**,** WCP).**

#### Mediating socio-economic crisis

During the COVID-19 pandemic lockdown, employed individuals who could support their families fared better than those who became unemployed. Those who maintained their jobs were able to retain some of their social connections, especially their colleagues.

I work as a networking specialist. So, I am doing a networking job. **(Voice 2; Malawi; Asylum seeker**,** GP).**

That was when I joined the Writing Centre as a consultant and tutor **(Voice 15; Zimbabwe; Permit Holder**,** WCP)**.

In many cases, employers, especially those in the private sector, withheld payment from their workers. However, some employers provide financial assistance to their staff.

My brother was getting assistance from the boss **(Voice 6; Zimbabwe; Undocumented**,** GP)**.

The teacher who supervises my work is the one who helped me a lot more **(Voice 14; Congo DRC; Asylum Seeker**,** WCP)**.

My boyfriend’s work gives them half their salary **(Voice 19; Zambia; Asylum Seeker**,** GP)**.

Financial assistance also came from families, friends, and loved ones, which helped the participants address some of their financial needs.

So, I have to call friends who are good supporters for us. **(Voice 1; Tanzania; Permit Holder**,** GP).**

Yeah. But the money has just been finished, and my brother has been helping me **(Voice 6; Zimbabwe; Undocumented**,** WCP).**

#### Outcome of mediating socio-economic crisis: financial survival

Most of the participants suggested that with the financial support they received from different sources, especially from family members, they were able to survive the COVID-19 pandemic era.

They [my family] give me some money to manage **(Voice 14**,** Zimbabwe; Undocumented**,** WCP).**

Part of my ability to stay here is because of remittances from my eldest brother **(Voice 14; Congo DRC; Asylum Seeker**,** GP)**.

Another participant suggested they could navigate the COVID-19 pandemic era because they became employed.

I was working in a medical organization. That is how I manage my life during lockdown **(Voice 10**,** Rwanda; Asylum Seeker**,** GP)**.

### Food insecurity as migrant precarity and resilience

**Fig. 3:**
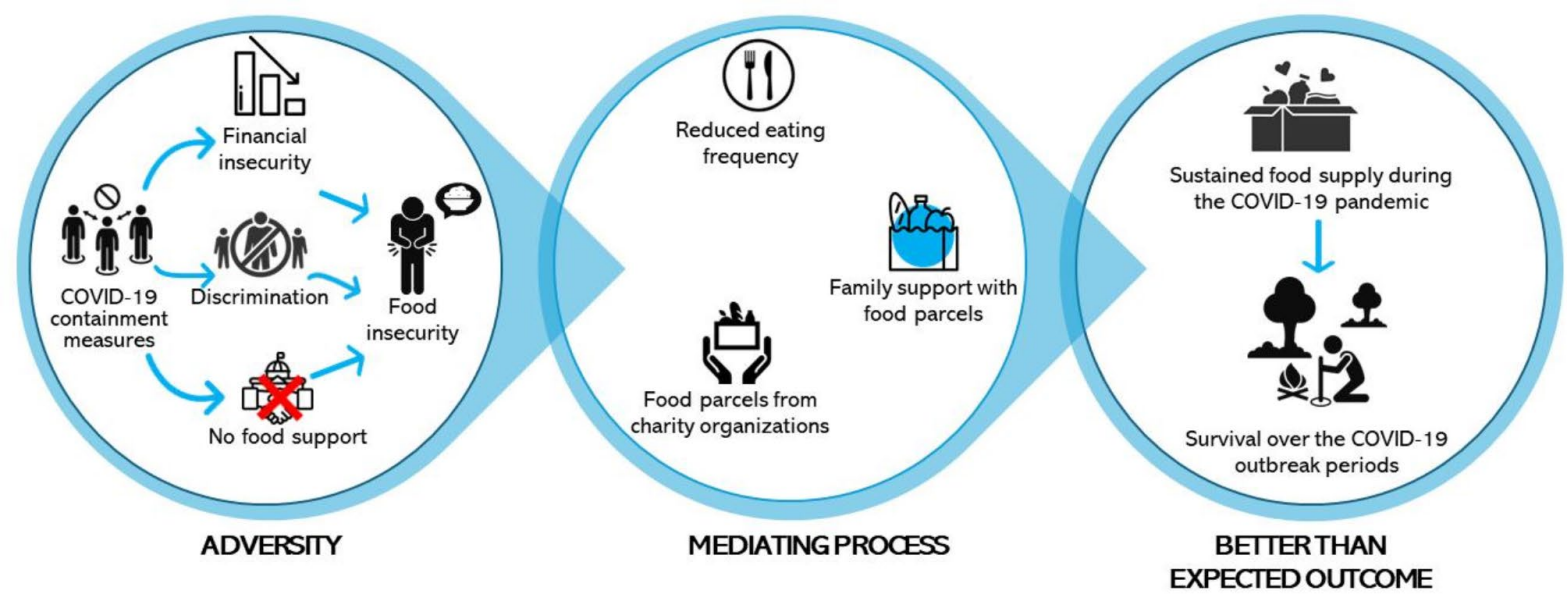
A resilient theory conceptualization of food insecurity based on emerging data

Food insecurity is one of the most significant consequences of the COVID-19 pandemic and associated preventive lockdown measures. Figure 3 shows our configuration of the emerging themes based on the resilient theory framework regarding food insecurity as the identified adversity.

The themes that established food insecurity as migrant precarity are related to financial insecurity, discrimination experienced during government relief programs, and poor access to food because of the lockdown. Following the food insecurity-related challenges facing the migrants, they embarked on some mediating behaviors, including reducing their eating frequency and receiving food parcel support from family and charity organizations. Following these mediation behaviors, migrants could push their available food supply through the COVID-19 era.

#### Food insecurities

as pertains to food insecurity as the identified adversity.

Participants reported not having access to sufficient food or food of adequate quality. Some of the participants acknowledged that it was a challenge to find what to eat.

The challenge was to get something to eat, especially my baby because I did not want to see him suffer. I do not want to see him growing up like I did. So, mostly, I go out of the way to sacrifice for him **(Voice 7; Zimbabwe; Undocumented**,** GP).**

Food insecurity was exacerbated because migrants did not receive any assistance from the South African government as South African citizens.

So, I still have not seen anyone who said they got a food parcel from the government **(Voice 10 Rwanda; Asylum Seeker**,** WCP)**.

We tried calling the [contact] numbers they provided, saying,' If you need something, dial it.’ We got nothing **(Voice 12; Malawi; Asylum Seeker**,** WCP)**.

Many of the COVID-19 pandemic relief programs implemented by the South African government exacerbated the perceived discrimination because they excluded most migrants.

But unfortunately, every time we apply, we cannot benefit. It benefited the South Africans more than the international students **(Voice 15; Zimbabwe; Permit Holder**,** WCP)**.

I do not even remember anyone [migrants] who benefited. Those who benefited may have been South Africans staying on campus. **(Voice 15; Zimbabwe; Permit Holder**,** WCP)**.

Competing priorities stemming from financial insecurity led to food insecurity. Some participants suggested that paying house rent was a priority over buying food.

There was a time when the landlord wanted the rent money, so we could not buy food **(Voice 1; Tanzania; Permit Holder**,** WCP).**

She said they were not saving then, and the goal was to pay the rent and food as there was not enough money **(Voice 8; Rwanda; Asylum Seeker**,** GP).**

#### Mediating food-insecurity

A necessary mediation approach adopted by migrants was to reduce their frequency of eating.

Yeah. It has affected our eating periods and our eating timing a lot. We used to have breakfast, lunch, and supper. So, we had to cancel the breakfast and then go for lunch and supper, so the pattern was changed **(Voice 3; Zimbabwe; Undocumented**,** GP).**

But at that time, the money was not circulating as usual. So, we used to eat once a day. Tomorrow, once a day like that **(Voice 4; Zimbabwe; Undocumented**,** WCP)**.

Migrants suggested that they received food support from some families and friends.

Many people were getting food from family and friends **(Voice 8; Rwanda; Asylum Seeker**,** GP)**.

Someone who could bring me food because there were other brothers and sisters. They were given to me by the window **(Voice 12; Malawi; Asylum seeker**,** GP)**.

Participants reported that they received food parcels from religious and non-governmental organizations.

Then we got some food from this church in Pretoria West. I think most of the owners are from Nigeria because they’re foreigners. So, they were not discriminating. We register when it is time. They will call you to come and get [a food parcel]. I think it was two times they gave us parcels, and then I got a voucher from them **(Voice 7; Zimbabwe; Undocumented**,** WCP)**.

But what I tried was a refugee organization we call Scalabrini. We tried and were served **(Voice 14; Congo DRC; Asylum Seeker**,** WCP)**.

According to the participants, one of the things that could have made the COVID-19 pandemic lockdown easy for them is the ability to work and be renumerated. s

Because I don’t save that much from my salary, I don’t save that much. So, I was lucky because our organization didn’t close **(Voice 12; Malawi; Asylum Seeker**,** GP).**

#### Mediating food-insecurity outcomes

Some migrants overcame food insecurity and fed themselves throughout the lockdown period and the COVID-19 pandemic.

I managed to feed myself because I worked in a medical organization. That is why I manage the COVID situation. That is how I manage life during lockdown **(Voice 10; Rwanda; Asylum Seeker**,** GP)**.

Some participants suggested that the food they received from different avenues allowed them to survive.

When they share their food, they give us [foreign migrants] a small amount. I survived that time **(Voice 5; Malawi; Asylum Seeker**,** GP)**.

### Health crisis as migrant precarity and resilience

**Fig. 4:**
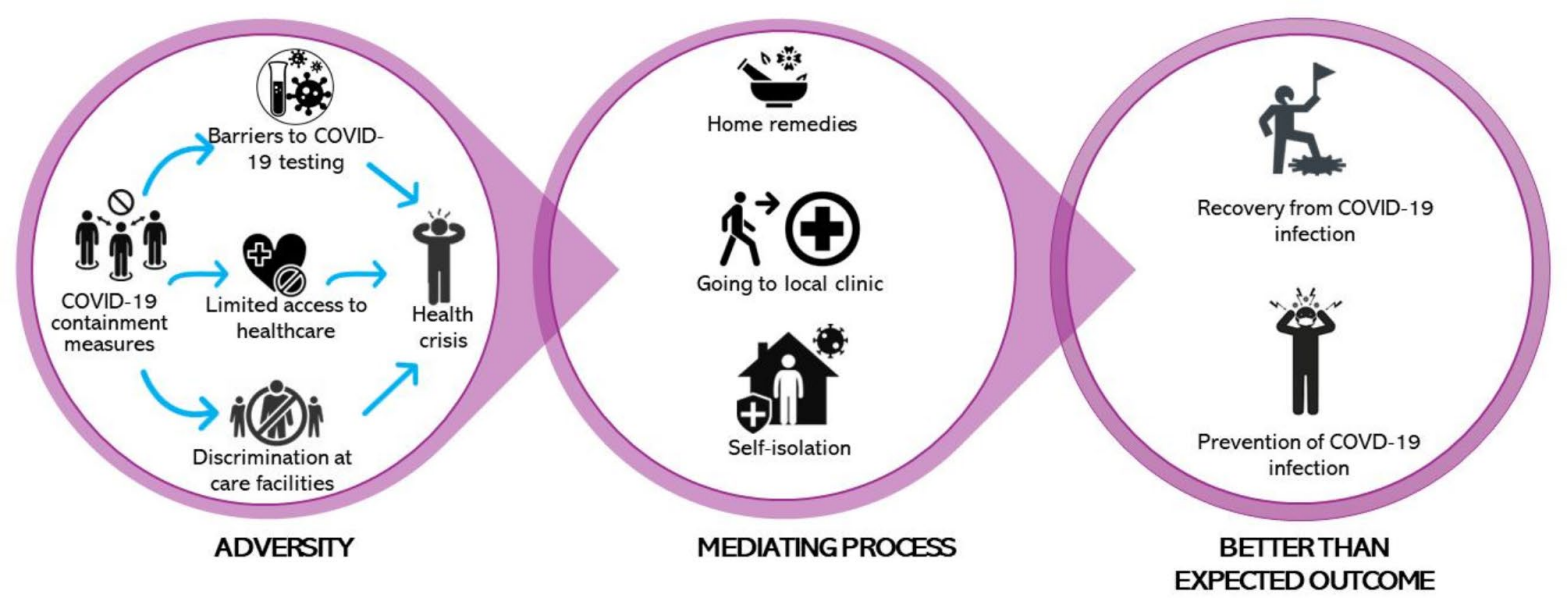
A resilient theory conceptualization of health crisis based on emerging data

A health crisis emerged as a third major theme in our data. Figure 4 shows that this crisis was caused by structural barriers that hindered access to COVID-19 testing, limited access to healthcare for those with possible COVID-19 symptoms, and discrimination at healthcare facilities.

Health-related precarity emanated from experiences of discrimination, which led to limited access to health care and COVID-19 testing and treatment services. The health precarity was mediated through home remedies, such as going to the local healthcare facilities, despite the perceived discrimination and the adoption of self-isolation. These activities lead to the prevention of COVID-19 (re)infection and recovery from COVID-19 infections.

#### Health-related adversities

Migrants in South Africa faced challenges in accessing healthcare facilities to manage COVID-19-related health issues. Some individuals reported instances of healthcare services being denied to migrants.

She said she went to that hospital from six o’clock until six o’clock in the afternoon, which ended that day. She never did anything. She went again the next day, and they had not even opened the file. I see that the hospital is filled with hatred because they don’t like us. I don’t want to go to that hospital (**Voice 8; Namibia**,** Asylum Seeker**,** WCP**).

It wouldn’t help if you went to Pretoria; they have hatred [for foreigners] there, and I don’t need to go to that side on the hospital day **(Voice 5; Malawi; Asylum Seeker**,** GP)**.

Some participants recounted their experiences of being infected with the COVID-19 pandemic.

I experienced a headache. A very painful headache. And my whole body was painful, and I was tired for the first two days. My two eyes couldn’t turn [SIC] like this was something different. **(Voice 12; Malawi; Asylum seeker**,** WCP)**

I became positive with COVID-19. I was isolating in my house **(Voice 3; Zimbabwe; Undocumented**,** WCP).**

Participants also alluded to being unable to afford the cost of COVID-19 tests when they felt they needed to take one.

I can’t say I had problems except for the R800 [approximately $60] for the [PCR] test when you’re just broke **(Voice 14; Congo DRC; Asylum Seeker**,** WCP)**.

Some participants suggested that social distancing associated with the lockdown disposed them to mental health issues.

My social life was a bit compromised, which affected me. And you know it will affect your mind. You cannot cope **(Voice 15; Zimbabwe; Permit Holder**,** GP)**.

They take the test like your blood pressure. If there is nothing to do with blood pressure, you end up saying okay, maybe what exactly are you thinking about? But somehow, I am depressed about the text. And so, I can say depression might have cribbed in my life **(Voice 6; Zimbabwe; Undocumented**,** WCP).**

#### Mediating health crisis

Regarding the management of COVID-19 symptoms, most migrants reported using homemade remedies to go to the hospital.

So, if you go to the hospital, you are more likely to die. At home, there were techniques that foreigners used to protect themselves rather than going to the hospital **(Voice 14; Congo DRC; Asylum Seeker**,** WCP)**.

Yes, we will tell you to boil lemon, garlic, and ginger, and then you use the blanket to cover yourself and inhale. Even though medicine recognizes this technique, it is not scientifically proven (**Voice 7; Congo DRC; Asylum Seeker**,** WCP)**.

Nevertheless, other participants continued accessing their local clinic or hospital when needed.

I always approached my nearest clinic so that they could check me as soon as possible if everything was fine with me **(Voice 10; Rwanda; Asylum Seeker**,** GP)**.

She [her migrant neighbor] went to the hospital, which was a private clinic **(Voice 14; Congo DRC; Asylum Seeker**,** WCP)**.

The migrants also practiced self-isolation or quarantine to prevent the spreading of the COVID-19 virus to those around them.

I became positive with COVID-19. I was isolating in my house. **(Voice 12; Malawi; Asylum seeker**,** WCP).**

#### Health mediating outcomes

According to the respondents, they utilized homemade remedies to prevent and alleviate their symptoms related to COVID-19.

We took precautions almost every week, like here at home, the ginger, as he just said we associated this with lemon and garlic. Every week, we boil this, and after we have a glass in the morning and a glass in the evening for maybe three days a week, it becomes a preventive remedy (**Voice 5; Zimbabwe; Asylum Seeker**,** WCP)**.

Citizens are strangers, and you can easily die there [at the hospital], and the techniques that we used [at home], many people said are effective. Many people around us used these techniques there because we were hesitant to go to the hospital, where there is discrimination. They would say, ‘he is a foreigner, and he can die. It is better to save our brother first’ **(Voice 14; Congo DRC; Asylum Seeker**,** GP).**

Other participants suggested that exercising, eating healthy, and sitting under the sun were helpful remedies for COVID-19 infections.

She [talking about his wife] was eating certain fruits. It would help if you exercised and sat under the sun for some time. All these things and the drinking of hot water were helping her. She Recovered **(Voice 18; Congo DRC; Asylum Seeker**,** WCP).**

## Discussion

This study examined the experiences of migrant populations in South Africa during the COVID-19 pandemic lockdown. The primary aim was to understand the difficulties encountered by migrants during the pandemic and their capacity to recover, as postulated in the resilient theory. By employing the IPA research approach and analysis, we conceptualize three critical areas of migrant pandemic precarity as adversities based on our resilient theory: socio-economic crisis, food insecurity, and health crisis. Our finding showed that these adversities relate to migrant precarity and transcend oft-discussed economic forms of precarity, illuminating some health implications. We also found (1) social support through family, work colleagues, and social service organizations; (2) corrective measures like reducing food portions and receiving food parcels; and (3) health-enhancing behaviors such as using traditional remedies, self-isolation and seeking healthcare as elements of the mediating process. Following the mediating process, these migrants could access finances, get food, and recover from COVID-19 symptoms. The resilient theory offered [40] a comprehensive view of migrant precarity and provided a heuristic tool to reveal the multidimensional, interrelated, and complex nature of overcoming the challenges posed by the COVID-19 pandemic on migrants [42]. Migrants demonstrated resilience by relying on mutual aid, making demands on the state and employers, and organizing private relief efforts [20]. As a result, we also investigated the processes by which these migrants overcome the challenges of the pandemic and attain outcomes that exceeded expectations using resilience theory.

One of the main areas of precarity migrants face in South Africa is the financial crisis. Our study found that restrictions, closure of activities, crumbling businesses, loss of jobs, and lack of savings led to an economic crisis for migrants in South Africa. Previous studies have also identified job loss or receiving low wages or no pay as a significant challenge during the COVID-19 pandemic lockdown [43–46]. What made the pandemic lockdown particularly difficult for migrant populations is that they typically work in the informal sectors, which were heavily affected by the lockdown measures [27, 47]. While our study identified other measures, such as receiving financial support from families, friends, and organizations, the ability to work (in-person, hybrid, and remote) was important for some migrants in overcoming the financial crisis. To reduce the financial inequities migrants face in South Africa, the government can enforce the crucial skills employment policy to have more migrants in critical jobs and those working remotely to earn income and mitigate their financial difficulties [48, 49]. A migrant with the requisite qualifications, work experience, and English language proficiency who falls into one of the specific critical skills categories stipulated by the Department of Home Affairs (DHA) of South Africa can be documented with the requisite critical skills visa or business visa. Although South Africa has policies to integrate migrants into their workforce, it has no proactive programs and mechanisms for profiling, targeting, attracting, and recruiting these critical skills [50]. Integrating more migrants with critical stills in the South African workforce will not only address the employment inequities faced by this population but also improve the economic growth of South Africa.

Our study found that food insecurity was a significant issue for migrants during the pandemic. A UN Refugee Agency report showed that by the end of May 2020, over 3,000 migrants had called their offices in South Africa, and over 95% of them had lost their source of income and faced hunger or eviction. Financial difficulties from losing paid jobs contributed to food insecurity [48, 51]. For some migrants, lack of proper documentation hindered their access to social support, including food parcels from the South African government, as documented in previous literature [27]. Even migrants with legal documentation were excluded from food parcel distribution due to their immigration status. While food insecurity affected many people in South Africa during the pandemic, migrants faced a challenging situation as they were not included in the government’s food program to mitigate the pandemic’s impact [27]. To address food insecurity during the COVID-19 lockdown measures, migrants in South Africa employed several strategies: (1) reducing their food portions or frequency of eating, (2) relying on support from friends, families, and employers, and (3) receiving assistance from religious and non-governmental organizations. These resilient mechanisms can be harnessed through the activities of civil society organizations on migration and refugee issues with state-making processes in South Africa [52]. Vanyoro [52] found that the non-profit migrant organization sector is weakened by ‘exploitative’ connections between NGOs and funders due to their financial vulnerability. NGOs should continue to leverage funds from international agencies like UNHCR, whose mandate is to help refugees and support their activism and assistance of migrants living in South Africa. Also, strengthening these connections by clearly defining concerns and priority categories, including addressing migrant food insecurities in pandemic conditions, can address migrant precarity. Studies have found that social support from social groups, especially religious organizations, is crucial in enhancing migrants’ resilience during the pandemic [53, 54].

Our study identified the health crisis as the third aspect of migrant pandemic precarity. Health-related precarity encompassed direct COVID-19 infection, difficulties in accessing preventive and curative resources, and mental health challenges. A study conducted on African communities in Australia confirmed that social isolation and financial insecurity resulting from joblessness or reduced working hours led to stress, frustration, anxiety, sadness, loneliness, and depression [54]. Other authors have also documented the social, occupational, and financial stressors associated with migrant pandemic precarity, which increase the risk of lower well-being [46, 53, 55]. Discrimination experienced by migrants in healthcare settings also contributed to their mistrust of healthcare providers and aid agencies in South Africa [56]. This distrust discouraged migrants from accessing COVID-19-related services, further exacerbating their healthcare-related precarity. Processes to mitigate health-related challenges include continuing to access local healthcare services, utilizing homemade remedies, and practicing self-isolation or quarantine when infected with COVID-19. While these health-resilient remedies were useful, they were certainly not sustainable. To ensure full health benefits among this population, the South African government must extend healthcare access to the entire immigrant population, regardless of their legal status. The South African Constitution guarantees migrants access to healthcare. The Constitution, Section 27 (1) (a) and (3), does not discriminate against anyone irrespective of nationality. Therefore, migrants have access to basic healthcare, including emergency medical treatment, as Sect. (3) of the Constitution indicates. Nevertheless, the attitudes of healthcare professionals suggest an unwillingness to provide healthcare services to undocumented migrants [57]. This challenge calls for healthcare professional training to address the challenge of access to healthcare services among the migrant population in South Africa.

### Strengths and limitations of the study

The study’s strength lies in its adoption of the IPA methodology, which allowed for a detailed examination of the personal experiences of migrants living in South Africa amidst the pandemic. This approach was valuable as the study occurred when South Africa implemented stricter COVID-19 restrictions to combat the third wave. Engaging directly with participants could capture their experiences in real-time, avoiding reliance on memory or preconceived notions.

Another strength of the study was using the resilient theory as a conceptual framework. This framework enabled us to systematically explore migrant pandemic precarity, mediation processes, and outcomes. A clear understanding was gained by analyzing how migrants utilized various resilient strategies from different sources to overcome challenges.

The maximum variation approach employed in sampling was also a strength. Using multiple recruitment methods and striving to include migrants from diverse conditions and backgrounds, a comprehensive range of experiences was captured, providing a holistic view of the migrant experience.

However, it is vital to acknowledge the study’s limitations. Firstly, the participants were only selected from two out of the nine provinces in South Africa due to limited funding. While South Africa has migrants in all provinces, the focus was restricted to the two provinces with the largest migrant populations. Despite this limitation, the findings from these provinces can be applied to the other seven provinces, given the contextual similarities in government laws and operations.

Another limitation is that the interviews did not include all relevant stakeholders. Although the study primarily focused on migrants’ experiences and their resilience during the COVID-19 pandemic, participants mentioned the influence of other stakeholders, such as discrimination at healthcare facilities. Therefore, including the perspectives of government officials, service providers, and community leaders would have enhanced the information obtained from migrants and allowed for data triangulation. This limitation highlights the need for further qualitative inquiry.

### Positionality

In the data collection section of this study, we noted that we adopted phenomenological reduction because we, the authors, are migrants living in South Africa. However, we have not fully experienced the precarity faced by all migrants. Both authors hold positions at higher education institutions in South Africa, and our study’s design and execution were influenced by concerns raised by other migrants in our social circles. Both authors were involved in data collection, preparation, and analysis. Throughout these processes, we reflected on our experiences during the COVID-19 lockdown and its challenges from a relatively privileged position. Our reflexivity aimed to enhance our phenomenological reduction and clarify our positionality, guiding readers in interpreting our findings.

## Conclusion

The concept of migrant pandemic precarity refers to the increased vulnerability of migrants during the COVID-19 pandemic, which is reported to be greater than that of the host country citizens. Despite facing adversity, humans often display resilience. In our study, we observed how migrants in South Africa successfully navigated migrant pandemic precarity and achieved better-than-expected outcomes during the COVID-19 pandemic lockdown. While resilience can arise from individual, family, and community sources, most protective factors originate externally (from family and community sources). To enhance the resilience of migrants during a pandemic, we recommend that the South African government should create mechanisms and processes to identify and integrate migrants with critical skills into their workforce, enhance collaborations between civil society organizations, local governments, and international organizations, such as the International Organization for Migration, to address food insecurities among the migrant population. Moreover, a pandemic-resilient planning framework should be adopted to address the pandemic precarity and adaptation practices of migrants, which may differ across different spaces and scales.

## Electronic supplementary material

Below is the link to the electronic supplementary material.

Supplementary Material 1

## Data Availability

Data is provided within the manuscript or supplementary information files.
