# Supplementary material for: Using the resilience theory to understand and address migrant pandemic precarity among South African migrant populations
Source: Arch Public Health. 2025 Apr 3;83:89. doi: 10.1186/s13690-025-01573-9 (PMC11969881; doi:10.1186/s13690-025-01573-9)
Supplement: Supplementary file 1 — Supplementary Material 1 [file 13690_2025_1573_MOESM1_ESM.docx]

**Using the resilience theory to understand and address migrant pandemic precarity among South African migrant populations**

**Financial and food insecurity questions**

- Describe how you manage to feed yourself and provide food for your family during the lockdown?
- Compare your eating pattern before and during the lockdown

Probe: How many times did you people manage to eat in a day?

- Describe the assistance that you received (food packages, vouchers etc) from any of the charity organizations or the government during the lockdown?
- Describe how NGO and government include asylum and refugee in their lockdown relief programmes
- What kind of help or support did you receive from any other person such as families, friends and neighbors? Probe: How often did you receive this help?
- Describe the kind of work you do before and during lockdown?
- Describe the type income you were receiving during the lockdown? Probe: Were you employed before and during the lockdown and at the moment?
- Describe the kind of savings (cash and kind) you have the lockdown (savings in your bank account before the lockdown)
- What made things difficult for you during the lockdown?
- Describe what would have make things easy for you doing the lockdown?

**Access to healthcare-related questions**

- Can you describe the symptoms of the Corona virus? Probe: Have you or anyone you know had any of those symptoms?
- Describe what you need to do and where to go if you have the symptoms?
- Describe how easy or difficult is it for you or anyone know to get tested for the Corona virus?
